# Combining BRAF inhibitor and anti PD-L1 antibody dramatically improves tumor regression and anti tumor immunity in an immunocompetent murine model of anaplastic thyroid cancer

**DOI:** 10.18632/oncotarget.7839

**Published:** 2016-03-02

**Authors:** Eran Brauner, Viswanath Gunda, Pierre Vanden Borre, David Zurakowski, Yon Seon Kim, Kate Virginia Dennett, Salma Amin, Gordon James Freeman, Sareh Parangi

**Affiliations:** ^1^ Department of Surgery, Massachusetts General Hospital, Harvard Medical School, Boston, MA, USA; ^2^ Departments of Surgery, Boston Children's Hospital, Harvard Medical School, Boston, MA, USA; ^3^ Departments of Anesthesia, Boston Children's Hospital, Harvard Medical School, Boston, MA, USA; ^4^ Department of Surgery, Ulsan University Hospital, University of Ulsan College of Medicine, Ulsan, Korea; ^5^ Department of Medical Oncology, Dana-Farber Cancer Institute, Harvard Medical School, Boston, MA, USA

**Keywords:** anaplastic thyroid cancer, programmed cell death-1, programmed cell death-ligand 1, BRAF inhibitor, MEK inhibitor

## Abstract

The interaction of programmed cell death-1 and its ligand is widely studied in cancer. Monoclonal antibodies blocking these molecules have had great success but little is known about them in thyroid cancer. We investigated the role of PD-L1 in thyroid cancer with respect to BRAF mutation and MAP kinase pathway activity and the effect of anti PD-L1 antibody therapy on tumor regression and intra-tumoral immune response alone or in combination with BRAF inhibitor (BRAFi). BRAF^*V600E*^ cells showed significantly higher baseline expression of PD-L1 at mRNA and protein levels compared to BRAF^*WT*^ cells. MEK inhibitor treatment resulted in a decrease of PD-L1 expression across all cell lines. BRAFi treatment decreased PD-L1 expression in BRAF^*V600E*^ cells, but paradoxically increased its expression in BRAF^*WT*^ cells. BRAF^*V600E*^ mutated patients samples had a higher level of PD-L1 mRNA compared to BRAF^*WT*^ (p=0.015). Immunocompetent mice (B6129SF1/J) implanted with syngeneic 3747 BRAF^*V600E/WT*^ P53^−/−^ murine tumor cells were randomized to control, PLX4720, anti PD-L1 antibody and their combination. In this model of aggressive thyroid cancer, control tumor volume reached 782.3±174.6mm^3^ at two weeks. The combination dramatically reduced tumor volume to 147.3±60.8, compared to PLX4720 (439.3±188.4 mm^3^, P=0.023) or PD-L1 antibody (716.7±62.1, P<0.001) alone. Immunohistochemistry analysis revealed intense CD8^+^ CTL infiltration and cytotoxicity and favorable CD8^+^:Treg ratio compared to each individual treatment. Our results show anti PD-L1 treatment potentiates the effect of BRAFi on tumor regression and intensifies anti tumor immune response in an immunocompetent model of ATC. Clinical trials of this therapeutic combination may be of benefit in patients with ATC.

## INTRODUCTION

Thyroid cancer is the most common endocrine malignancy [1]. Clinical outcomes vary depending on the pathologic subtype, of which there are four: papillary (PTC), follicular (FTC), medullary (MTC), and anaplastic (ATC). Differentiated tumors (papillary or follicular) are highly treatable and usually can be cured. Poorly differentiated, medullary or anaplastic tumors are much less common, more aggressive, metastasize early, and have a far poorer prognosis. While the majority of patients respond well to surgical resection, a significant number suffer from recurrence and some patients have an aggressive course and present with locally advanced disease or metastasis [2]. Anaplastic thyroid cancer (ATC) is one of the worst human malignancies, associated with median survival of only 2-3 months [3]. As the mutational landscape of thyroid cancer is being better defined through recent large-scale efforts by the NIH Human Genome Atlas Project, as well as by individual labs, targeted therapies aimed at these mutations are entering the clinical environment [4, 5].

In the current era of personalized medicine, strategies that exploit the mutational profile of individual tumors have come to the fore. Recent work in both preclinical studies and clinical trials has focused on the known driver mutations in thyroid cancer. Altered MAPK signaling pathway in many thyroid cancers leads to ERK phosphorylation, proliferation, and invasion hence BRAF inhibitors (BRAFi) and MEK inhibitors (MEKi) have been the subject of intense investigation. Various inhibitory molecules have been tested, including vemurafenib (PLX4032), which demonstrated a reasonable response in patients with progressive metastatic, radio-iodine refractory BRAF-positive PTC [6], but the response was relatively short-lived in an anecdotal report of patients with ATC [7]. Unfortunately, resistance to BRAFi therapy has proved to be a significant clinical obstacle in treating patients with melanoma, where it appears to lead to reactivation of the MAP kinase pathway within several months [8, 9]. Although few patients with aggressive thyroid cancers, such as ATC, have been treated with BRAFi therapy, resistance is expected to develop in these patients as well.

An alternative approach involving immune therapy, which previously had been rejected by most physician scientists as ineffective and cumbersome, has regained favor as a result of new knowledge about the immune checkpoint receptor, PD-1 and its ligand, PD-L1, which regulate T-cell activity in the tumor microenvironment. A role of the immune checkpoint regulators is to prevent autoimmunity and to reduce collateral tissue damage by modulating the immune response in chronic infection. Cancer cells capable of up regulating the expression of PD-L1 have the potential to become immune resistant [10], thereby evading destruction by T cells. The initial foray into the study of immune checkpoint regulators started with descriptions of CTLA-4 as a co inhibitory receptor. Since then several other immune checkpoint regulators have been identified [11]; the most widely studied has been the interaction between programmed death 1 (PD-1), a T cell co-inhibitory receptor, and its ligand, programmed death ligand 1 (PD-L1) [12].

PD-L1, also known as CD274, is a cell-surface glycoprotein of the B7 family. It is expressed on various tissues including solid tumors and can facilitate immune evasion and T cells exhaustion [13]. The interaction of PD-1 on the surface of T cells with PD-L1 on tumor cells, suppresses TCR-mediated proliferation and activation, and inhibits T cell cytolysis. As a result, increased PD-L1 expression by cancer cells is a fundamental host immune escape mechanism [14]. Many tumor cells increase PD-L1 expression in response to local factors such as Interferon-γ (IFN-γ) released from CD4 helper, activated CD8^+^ T cells or NK cells in the tumor microenvironment. Indeed, human tumors show a correlation between PD-L1 levels and T cell infiltration reflecting an ongoing but unsuccessful anti-tumor immune response. PD-L1 has been investigated as a potential marker for tumor aggressiveness [15, 16], and anti PD-1 and anti PD-L1 therapies in lung, renal cancer and melanoma are showing promising results [17, 18]. The critical role of PDL1-PD1 interaction has been shown in both preclinical models and clinical trials in many solid cancers but its role in thyroid cancer has been scarcely investigated and mostly in *ex vivo* experiments. [19–24]

Our study was designed to advance current understanding of the role of PD-L1 in thyroid cancer cells, thereby paving a path for future testing of PD-L1-based therapies in thyroid cancer patients. It is the first study to look at the expression profile of PD-L1 in a panel of nonmedullary thyroid cancer cells at baseline, after IFN-γ stimulation, and after treatment with MAP kinase inhibitors. It also represents the first attempt to determine the impact of PD-L1 antibodies, alone or in combination with BRAF inhibitor, on tumor volume in an *in vivo* immunocompetent murine model of anaplastic thyroid cancer. In undertaking this study, we hypothesized that PD-L1 expression in non-medullary thyroid cancer would correlate with MAP kinase signaling pathway activity, and as a result, targeted therapies that reduce MAP kinase activity, such as BRAFi and MEKi, would be found to regulate PD-L1 expression in BRAF-mutated tumors. We further proposed that blocking the interaction between PD-L1 and PD-1 with an anti-PD-L1 antibody, would have the added effect of increasing the anti-tumor activity of BRAFi-induced infiltrating T cells. In the first phase of our investigation, we studied 5 human and 4 murine thyroid cancer cell lines to determine baseline expression of PD-L1. Next, we investigated the effect of manipulating MAP kinase activity on PD-L1 expression *in vitro* and *in vivo.* Finally, we tested the effect of combining BRAFi and anti-PD-L1 antibody on tumor regression and intra-tumoral immune response in an orthotopic immunocompetent mouse model of ATC.

## RESULTS

### Thyroid cancer cell lines with the BRAF^*V600E*^ mutation express higher baseline levels of PD-L1 mRNA compared with BRAF^*WT*^

**Figure 1:**
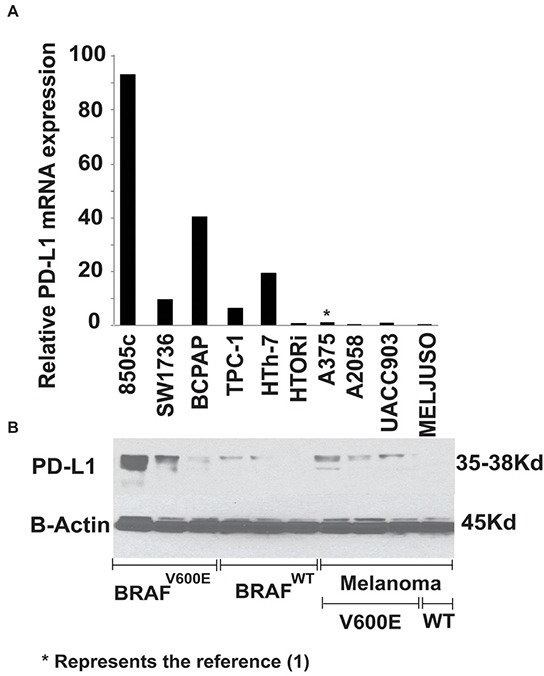
PD-L1 mRNA A. and protein B. expression of different human thyroid and melanoma cell lines The graph is representative of 3 different experiments using technical triplicate and is plotted as fold change normalized to the expression of A375. PD-L1 mRNA expression of BRAF^*V600E*^ (8505c, BCPAP, SW1736) thyroid cell lines showed higher base line expression compared to BRAF^*WT*^ cell lines (TPC-1, HTh-7 and the normal thyroid cells HTORi) (Mann-Whitney U, P≤0.05), thyroid cells as a group showed higher base line expression compared to 4 melanoma cell lines (A375, A2085, MEL-Juso and UACC-903) (Mann-Whitney U, P≤0.05). Western blot (B) analysis showed protein levels corresponded to mRNA levels in thyroid cells.

Given the known therapeutic implications of PD-L1 expression in melanoma, PD-L1 mRNA levels in 6 human thyroid cancer cell lines was quantified against 3 BRAF^*V600E*^ melanoma cell lines (A375, A2058 and UACC903) and one BRAF^*WT*^ (MelJuso) (Figure 1). BRAF^V600E^ mutant thyroid cancer cell lines showed significantly higher baseline expression of PD-L1 than the BRAF^V600E^ mutant melanoma cell lines; with 8505c cells showing the highest expression at 93-fold compared with A375 melanoma cells. Thyroid cell lines with the BRAF^*V600E*^ mutation also showed significantly higher baseline expression of PD-L1 mRNA compared with BRAF^*WT*^ thyroid cells (P<0.05). In fact, the normal HTORi cell line had the lowest expression of PD-L1. Western blot analysis pointed toward higher PD-L1 protein expression in the mutant BRAF cells compared with wild type across all cell lines investigated.

### BRAF^*V600E*^ mutated PTC tumors from patients showed higher PD-L1 expression compared to BRAF^*WT*^ tumors

**Figure 2:**
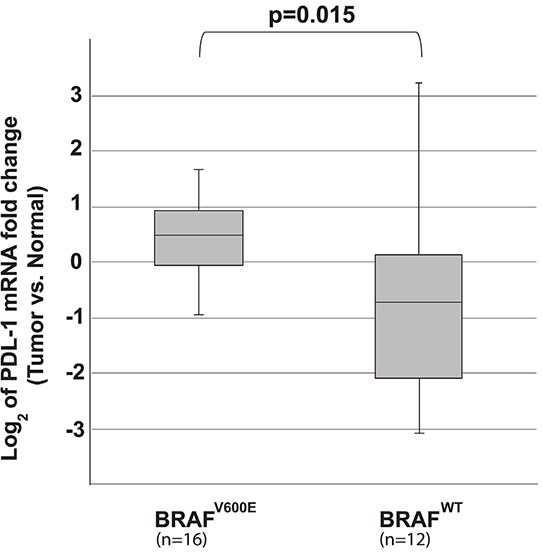
The log_2_ of the PD-L1 mRNA fold change of tumor vs. adjacent normal tissue in 28 patients Patients samples carrying BRAF^*V600E*^ (n=16) showed higher induction of PD-L1 compared to BRAF^*WT*^ tumors (n=12) (medians: 0.51 vs. −0.70, P = 0.015).

To determine whether tissue from patients with BRAF^V600E^-mutated tumors also expressed higher levels of PD-L1, PD-L1 mRNA expression levels were analyzed in randomly selected 28 fresh frozen PTC tumors and their matched normal thyroid tissue samples. Fifty-seven percent had BRAF^*V600E*^ mutations on routine sequencing. None of the demographic or tumor characteristics were significantly different between the BRAF^*V600E*^ (n=16) and the BRAF^*WT*^ (n=12) groups (Table 1). PD-L1 mRNA expression levels in the normal thyroid were set to one for analysis purposes, and log2 of fold changes was used for plotting the box graph. The BRAF^*V600E*^-mutated tumors showed significantly higher expression of PD-L1 mRNA compared to BRAF^*WT*^ tumors (log2 of fold changes: median (inter quartile range): 0.51 (−0.05 to 1.04) vs. −0.70 (−2.24 to 0.28) (P = 0.015) (Figure 2).

**Table 1:** Patient and tumor characteristics

	*Total n=28*	*BRAF^V600E^ n=16*	*BRAF^WT^ n=12*	*P value*
***Female Sex (%)***	16 (57)	10 (63)	6 (50)	0.70
***Age [year](range)***	53 (29-84)	53 (35-84)	51 (29-76)	0.88
***Tumor size (%)***				0.45
***T1-2***	15 (54)	10 (63)	5 (42)	
***T3-4***	13 (46)	6 (37)	7 (58)	
***Lymph node status (%)***				1.00
***Nx/N0***	18 (64)	10 (63)	8 (67)	
***N1***	10 (36)	6 (37)	4 (33)	
***TNM stage (%)***				0.53
***1***	7 (25)	4 (25)	3 (25)	
***2***	2 (7)	1 (6)	1 (8)	
***3***	13 (47)	9 (56)	4 (33)	
***4***	6 (21)	2 (13)	4 (33)	

### PD-L1 expression in human thyroid cancer cell lines is dependent on MAP kinase pathway activation

**Figure 3:**
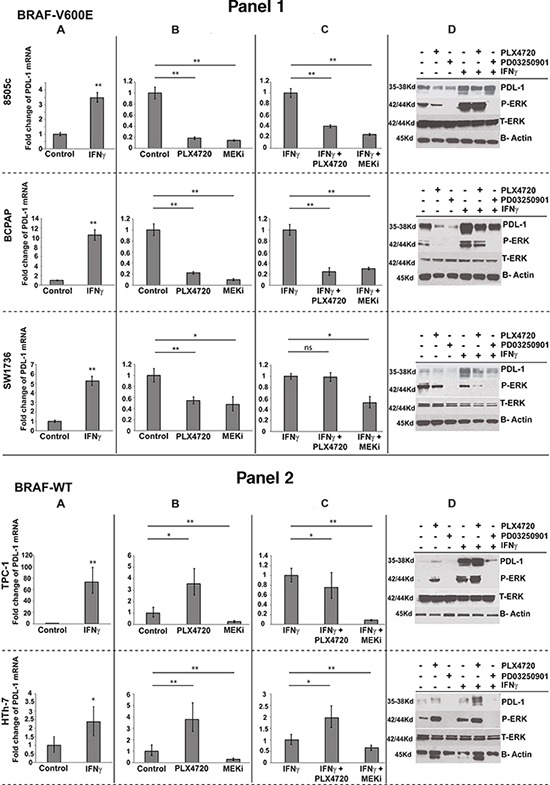
Five human thyroid cancer cell lines were treated for 24 hrs with A IFNγ. alone **B.** PLX4720 or PD03250901 alone or in combination with IFNγ **C.** Quantitative PCR fold change of PD-L1 mRNA was normalized to control (A, B) or to IFNγ treatment state (C). **D.** Western blot analysis for PD-L1, total ERK, phospho ERK and β-actin were done for all treatment combination mentioned above (* indicate p<0.01, ** indicate p<0.001, ns- not significant).

Generally, infiltrating T cells in the tumor stroma produce IFN-γ, which is a potent inducer of PD-L1 expression on tumor cells, thus potentially diminishing the anti-tumor immune response [25–27]. In our thyroid cancer cell line panel, IFN-γ appropriately increased both mRNA and protein expression of PD-L1 in all cell lines (Figure 3A).

To assess whether MAP kinase pathway activity alters PD-L1 expression, we measured PD-L1 expression in our thyroid cancer cell line panel after treating with BRAFi PLX4720 or MEKi PD0325901. All cells responded appropriately to MEK inhibition, showing a decrease in pERK. Similarly, BRAF inhibitor decreased pERK in cells with BRAF^*V600E*^ mutations (Figure 3 Panel 1,D), but as expected, caused a paradoxical increase in pERK in the BRAF^*WT*^ cancer cells (Figure 3 Panel 2,D) [28]. Both pERK and PD-L1 both mRNA (Figure 3B) and protein (Figure 3D) expression were significantly reduced in all cells treated with MEKi, regardless of mutation status. In contrast, when treated with BRAFi, mutation status of the cell was important in determining the changes in PD-L1 expression. While treatment with BRAFi caused significant decreases in both pERK and PD-L1 expression in BRAF^*V600E*^ cells (Figure 3 Panel 1B, D), the BRAF^*WT*^ cells treated with BRAFi showed the expected paradoxical increases in pERK which was accompanied by increases in PD-L1 expression (Figure 3 Panel 2B, D). BCPAP showed the largest reduction when exposed to MEKi in comparison with untreated control, with a 10-fold decrease of PD-L1 expression, and 8505 cells showed a 6.9-fold decrease. Western blot analysis demonstrated tight correlation between the mRNA and protein expression of PD-L1 through the different treatments and tight correlation of PD-L1 protein expression with MAP kinase phosphorylation status (Figure 3D).

When cells were treated in the context of stimulation by IFN-γ, similar trends were seen depending on the mutation status of the cells. Treatment with MEKi in the presence of IFN-γ resulted in similar impressive decreases in PD-L1 mRNA and protein expression in all cell lines regardless of mutation status (Figure 3C, D). Treatment with BRAFi in the presence of IFN-γ resulted in significant decreases in PD-L1 mRNA and protein levels in two of the BRAF^V600E^-mutated cell lines (8505 and BCPAP), whereas in the SW1736, only decreases in PD-L1 protein expression were observed. In BRAF^*WT*^ cells treated with PLX4720 in the presence of IFN-γ, consistent increases were only observed in the protein levels of PD-L1. Significant increases in PD-L1 mRNA were observed only in the HTh-7 (ATC) cell line (Figure 3C, D).

### MAP kinase pathway inhibition decreases PD-L1 expression in human thyroid cell lines *in vivo*

**Figure 4:**
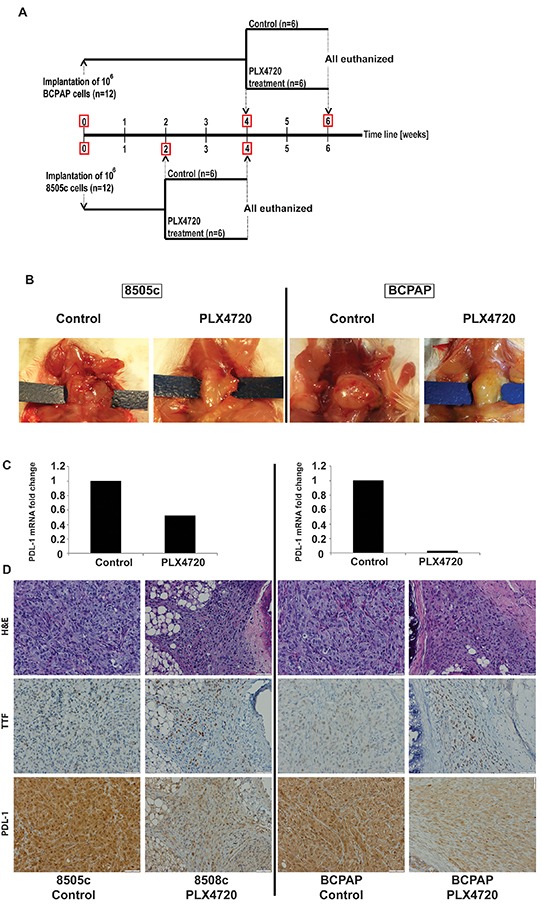
Schedule for the experiment using Orthotropic SCID mouse model for 2 human thyroid cancer cell lines (8505c; ATC derived, and BCPAP; PTC derived cells) is outlined in **A.** In vivo results of PLX4720 treatment for both cell lines showing obvious tumor volume reduction **B.** PD-L1 mRNA expression of tumors pooled for each group showing significant reduction of PD-L1 (2 and 37 fold for 8505c and BCPAP respectively) when treated with PXL4720. **C.** IHC analysis for human PD-L1 showed a marked reduction of staining with PLX4720 treatment for both cell lines **D.**

To test whether BRAF inhibition also causes a decrease of PD-L1 expression *in vivo*, SCID mutant mice bearing 8505c and BCPAP orthotropic tumors were treated with BRAF inhibitor for two weeks, and PD-L1 mRNA and protein expression levels were subsequently measured (Figure 4A). Treatment with PLX4720 significantly reduced *in vivo* expression of PD-L1 mRNA in both tumor models; two-fold for 8505c and 37-fold for BCPAP. PD-L1 protein levels as measured by immunohistochemistry also showed a significant decrease in both tumors (Figure-4B).

### BRAF inhibition does not regulate PD-L1 expression in BRAF^*V600E*^ murine thyroid cancer cell lines

**Figure 5:**
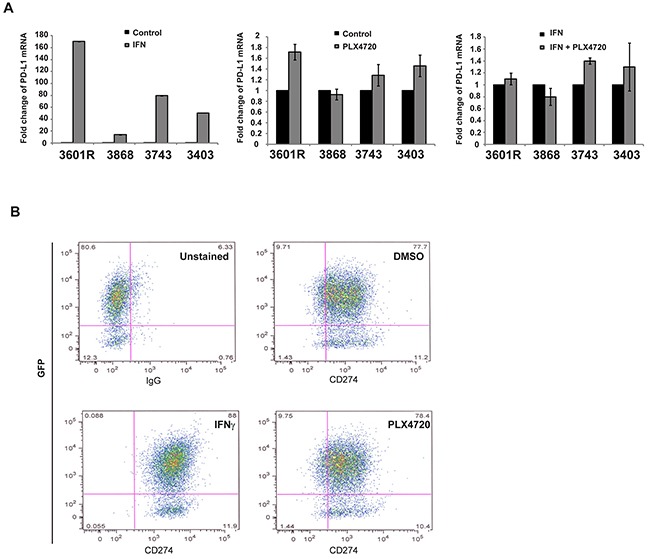
Murine cell lines were treated with PLX4720, IFNγ and the combination of the two PD-L1 mRNA expression was measured against GAPDH expression and compared to non treated cells. While IFNγ robustly increased PD-L1 mRNA expression in all cell lines, none of the four cell lines exhibited a significant change in PD-L1 expression (<2 fold change) when treated with PLX4720 (compared to control) or with the combination of IFNγ + PLX4720 (compared to IFNγ alone) **A.** Cell surface analysis of PD-L1 using FACS showed a saturation of PD-L1^+^ cells while PLX4720 treatment did not differ from DMSO alone. The same result was seen for all four murine cell lines. A representative of one cell line (3743) is shown **B.**

We checked the PD-L1 mRNA expression of genetically engineered murine thyroid cancer lines (3601R, 3868, 3743 and 3403) at baseline and in response to PLX720, IFN-γ, or both. Although IFN-γ caused a dramatic increase of PD-L1 expression; 3601R (93.7-fold), 3868 (13.9- fold), 3743 (74.8-fold), 3403 (20.2-fold) as compared to untreated control, none of the four cell lines showed significant (>2-fold) change in PD-L1 expression in the presence of BRAFi (Figure 5A), regardless of the presence of IFN-γ. Cell surface analysis of PD-L1 protein expression using FACS was consistent with this observation (Figure 5B).

### Combination of anti-PD-L1 antibody with PLX4720 was highly effective in reducing tumor volume in an immunocompetent orthotopic mouse model of thyroid cancer

**Figure 6:**
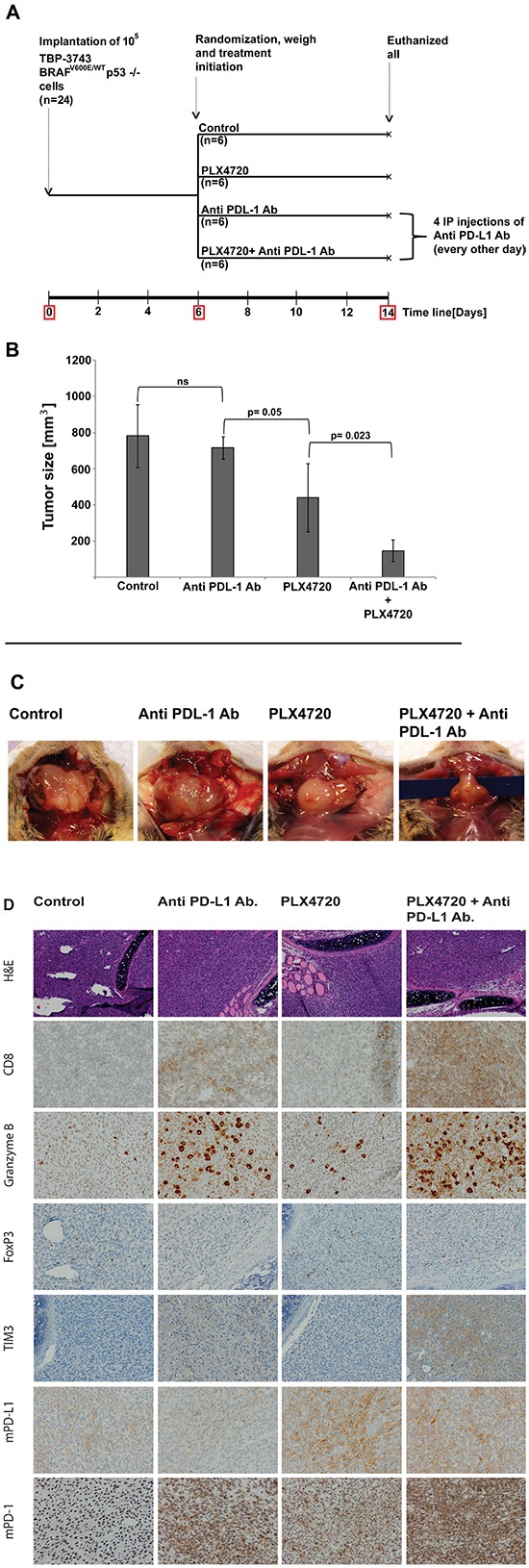
Schedule for the experiment using immunocompetent orthotropic mouse model implanted with murine cells 3473 **A.** TBP: ***T**POCreER; **B**raf ^tm1Mmcm/WT;^ Tr**p**53^tm1Brn/tm1Brn^* (TBP). IP: intraperitoneal. Tumor volume represents in cubic millimeters as mean±SD. The combination of PLX4720 and Anti PD-L1 monoclonal antibody had a dramatic effect on tumor volume reduction compared to control and to either treatment alone (147±61g, P<0.05) **B, C.** The presence of CD8^+^-CTL, the cytotoxicity marker; Granzyme B, Treg marker; FoxP3, T cell exhaustion marker; TIM3 and the expression of mouse PD-L1 and mouse PD-1 were analyzed using IHC. CD8^+^-CTL infiltration and cytoxicity were evident with anti PD-L1 treatment alone and were intensively stained when combined with PLX4720. The exhaustion markers TIM3 and PD-1 were evident with anti PD-L1 treatment but were highly induced when combining with PLX4720 **D.**

We used our previously described immunocompetent mouse model of ATC in which 10^5^ genetically engineered murine ATC; TBP 3743 BRAF^*V600E/WT*^ P53^*−/−*^ cells are implanted into an immunocompetent mouse thyroid, resulting in a very rapidly enlarging tumor with marked infiltration of CD8+ T lymphocytes when treated with PLX4720 [29]. Given the rapid nature of this model (life expectancy of control animals is two weeks), one week after tumor implantation, the mice were randomized to treatment with either anti-PD-L1 Ab, PLX4720, or a combination of both agents (see schema in Figure 6A). All animals were euthanized and tumor volume was measured after 8 days of treatment (i.e., 14 days post-implantation). Control animals had a median tumor volume of 782±175 mm^3^. Treatment with anti-PD-L1 Ab alone did not significantly reduce tumor volume (717±62mm^3^, p=1.00), whereas PLX4720 treatment resulted in significant tumor volume reduction (439±188 mm^3^, 44% tumor reduction, P = 0.007). Using the Wald chi-square for statistical analysis showed that the combination of PLX4720 and anti-PD-L1 antibody acted synergistically, and was the most effective in reducing tumor volume compared to all other treatment groups (147.3±60.8 mm^3^, showing a dramatic 81% tumor volume reduction compared to control (P < 0.001) and impressively over 66% better than those treated with PLX4720 alone (P=0.023) (Figure 6B, 6C).

### Blockade of the PD-L1 – PD-1 axis resulted in increased CD8^+^ T-cell infiltration and improved the cytotoxic profile of the T cells

To investigate the influence of anti-PD-L1 treatment alone and in combination with BRAFi on T-cell infiltration, the treated tumors from the immunocompetent mice were immunostained for CD8; TIM3, a marker of CD8^+^ cell exhaustion; and Granzyme B, a marker of T-cell cytotoxicity. Tumors from animals treated with PD-L1 blockade or PLX4720 alone had visibly higher infiltration of CD8^+^ T cells compared to controls; in the tumors treated with the BRAF inhibitor most of the T cells appeared to have infiltrated into the peripheral aspects of the tumor. Combining both drugs, however, dramatically increased CD8^+^ T-cell infiltration throughout the tumor (Figure 6D). Granzyme B staining is thought to show T cells involved in acute cytotoxicity, and again, the Granzyme B staining was most dramatically increased in the combinatorial treatment (Figure 6D). Patterns of T cells showing expression of TIM3 exhaustion markers were similar, with the highest levels of exhausted T cells seen in the combinatorial treatments.

T-regulatory cells (Tregs) have been shown to selectively interfere with the release of cytolytic granules by cytotoxic T lymphocytes (CTLs) in a reversible and TGFβ-dependent manner, thereby attenuating CTL-mediated cytotoxicity. Previous studies have shown that the CD8:Treg ratio is an important indicator of therapeutic benefit in melanomas, where potent synergistic effects of combination therapy using BRAFi and PD1 blockade cause increased CD8^+^:Treg ratio and enhanced cytokine production, compared with BRAFi alone in both mice and humans [30]. Indeed, in our model as well, we found that while PLX4720 alone appears to increase the infiltration of CD8+ T cells into the periphery of the tumor, unfortunately there was also a moderate increase in FoxP3 T regulatory cells. However, the combination of BRAFi and PD-L1 antibody yielded an obvious increased CD8^+^:Treg ratio in the tumor, which should be beneficial (Figure 6D).

The baseline expression of mouse PD-L1 (mPD-L1), as seen in the IHC staining of the control group, was observed to increase dramatically upon treatment with PLX4720 alone; while treatment with anti-PD-L1 antibody alone or in combination with BRAFi reduced the IHC staining expression of mPD-L1 as compared with control and BRAFi alone, respectively.

## DISCUSSION

During the last decade, most thyroid cancer therapeutics have targeted known oncogenic mutations, such as BRAF^*V600E*^, in an effort to alter thyroid cancer progression. Unfortunately, the response to these efforts to inhibit the MAP kinase pathway has been relatively short-lived as a consequence of the development of resistance to treatment. Coincident with this work, immune therapy, has regained favor as a result of new knowledge about the immune checkpoint receptor, PD-1 and its ligand, PD-L1, which regulate T-cell activity in the tumor microenvironment. In particular, studies involving the use of monoclonal antibodies directed against the PD-1 receptor and its ligand PD-L1 have shown promising response rates, leading to FDA approval of PD-1 drugs for advanced melanoma, lung and renal cancer [31, 32]. One exciting application of this approach is being tested clinically to determine which combinations of BRAF or MEK inhibitors, coupled with immunotherapy, will generate the more sustained tumor response.

In melanoma patients, the initiation of BRAFi therapy causes rapid T-cell infiltration into tumor, but the T cells are rendered ineffective after two weeks, probably resulting from a surge of expression of PD-L1 on the tumor cells [33]. This finding suggests that PD-1 pathway blockade might prolong the immune response. Moreover, by combining BRAFi and PD-1 pathway blockade, it might be possible to significantly augment and prolong the therapeutic response to BRAF inhibitors.

This approach might also be the logical next step in thyroid cancer, given the deregulation of the MAP kinase pathway in many aggressive thyroid cancers, but little is known about the baseline characteristics of these immune regulators in thyroid malignancy. Furthermore, as clinical trials in other malignancies mature, it is becoming increasingly clear that toxicity is a major barrier to treatment. Additional preclinical data providing mechanistic insight into the possible beneficial synergistic and toxic effects of these combinations in the various tumor components is essential.

The presence of PD-L1 in thyroid tumors was first described in 2003 by Brown et al. [24]. Subsequently, the role of PDL1-PD1 interaction in thyroid cancer has only been investigated in a handful of in vitro experiments and through staining of patient tissue samples; Cunha et al demonstrated higher levels of PD-L1 in papillary thyroid cancer tissue samples compared to normal and benign lesions and showed a positive correlation between PD-L1 levels and infiltration of CD8^+^, CD4^+^, FoxP3^+^ lymphocytes and tumor associated macrophages [19, 20]. French and colleagues found PD-1^+^ T lymphocytes to be enriched in metastatic lymph nodes especially in recurrent disease and were markedly associated with extra nodal invasion [21]. Interestingly, this same group recently suggested that tumor associated lymphocytes, taken from grossly involved lymph nodes, exhibit incomplete exhaustion which may thus be reversible [22]. Given that in patients, the immune system itself does not appear capable of actively engaging and destroying metastatic deposits in lymph nodes, testing combinations of therapeutics that inhibit the suppressive activity of Tregs may be valuable. The MAP kinase pathway-dependent regulation of PD-L1 in thyroid cancer was implied when a positive correlation between BRAF^*V600E*^ and high level of PD-L1 in 33 PTC samples was recently reported [23]. Both BRAF and PD-L1 expression was tested in 13 patients with ATC, 3 patients were found to have BRAF^*V600E*^ mutations, and 3 had high expression of PD-L1, there was one patient with both [34].

In this study, we have established that thyroid cancer cell lines and tumor samples from patients with BRAF^*V600E*^ -mutated tumors have higher levels of PD-L1 compared with either BRAF^*WT*^ tumors or matched normal tissue. Additionally, we have validated the combination of PD-L1 blockade and BRAF-targeted therapy in a novel immunocompetent model of ATC using rapidly growing genetically engineered BRAF^*V600E*^-mutated tumors. Our model is clinically relevant on multiple fronts. First, the genetically engineered cell lines have both BRAF and p53 mutations allowing for treatment to be initiated when the tumors are large and still rapidly growing. Second, the immune competent nature of our model permits us to study the immune milieu inside the tumor. Our studies did show that PD-L1 blockade has very minimal effects on the tumor when given alone, despite fairly impressive infiltration of CD8^+^ T cells and high activity of Granzyme B. This finding is not entirely unexpected, however, given the aggressiveness of this specific ATC model in which more conventional agents such as BRAF or Src inhibitors were less effective [35]

In contrast, the combination of PD-L1 antibody and BRAF inhibitor produced a powerful synergistic response. The synergistic improvement in tumor shrinkage in those animals treated with a combination of BRAFi and PD-L1 antibody was also associated with an increase in tumor-infiltrating lymphocytes (TIL) and TIL function. The mechanisms underlying TIL infiltration were not studied here. It could be the result of clonal expansion or increased trafficking of T cells into the tumor. Previous studies in melanoma animal models and patient trials indicate that both mechanisms are likely at play [33]. Clearly, combined therapy with BRAFi and PD1 blockade caused an increase of the CD8+:Treg ratio, which favors cytokine production and destruction of tumor cells. In fact, the observed increase of Granzyme B activity, signaling increased T-cell cytotoxicity, in tumors treated with combination therapy also supports this view. Our results are consistent with several other published studies showing synergy of BRAF inhibitors with other immunotherapies in murine models [36, 37].

Little is known about the effects of immune checkpoint regulators in thyroid cancer. The therapeutic effect of blocking either PD-1 or PD-L1 previously has not been tested. PD-L1 is expressed by multiple cell types in tumors, thus underscoring the importance of these *in vivo* studies. PD-L1 expression in lymphoid organs may suppress the initial activation of T cells or perhaps even induce Tregs. PD-L1, expressed on both tumor and non-tumor cells has the potential to suppress antitumor immunity by contributing to T-cell exhaustion.

**Figure 7:**
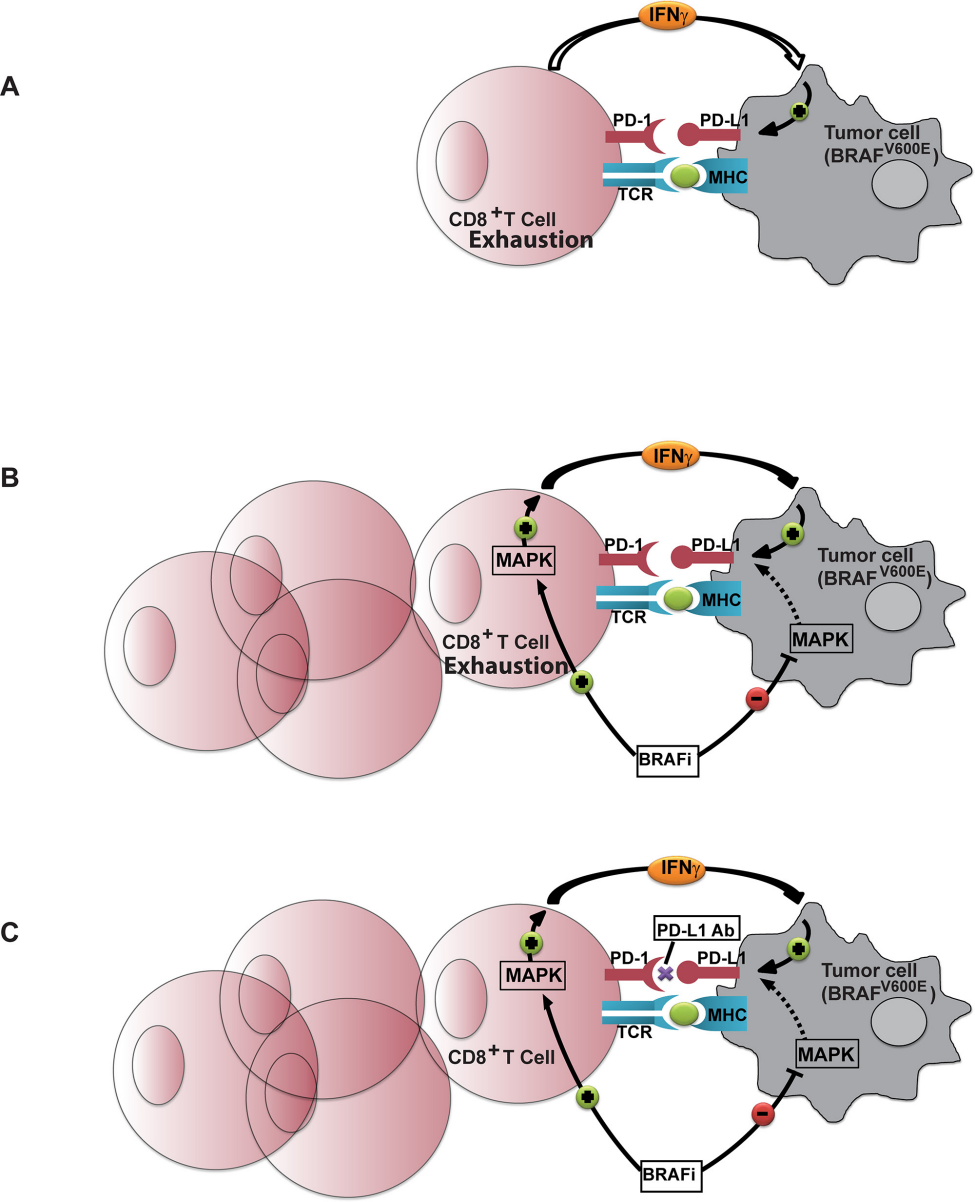
The rational for adding Anti PD-L1 monoclonal antibody to BRAFi treatment **A.** In the naïve environment the lack of migration of T-cells into the tumor site and the mild reactivity that drives T-cells to produce cytokine (among them IFNγ) has substantial impact on the expression of PD-L1 which tempers the already weak anti tumor reaction. **B.** BRAFi treatment results in increased T cell infiltration into the tumor microenvironment. Although BRAFi potentially down-regulates the expression of PD-L1 on BRAF^*V600E*^ tumor cells by down regulating MAP kinase activity in these cells, it paradoxically up-regulates MAP kinase in the BRAF^*WT*^ T-cells which results in IFNγ production and intensive up-regulation of PD-L1 on tumor cells. Immune response with BRAFi alone therefore does not come to its full potential. **C.** Adding anti PD-L1 antibody is an additional step necessary to allow the immune response facilitated by BRAFi to reach its full potential.

Treatment with MAP kinase inhibitors, such as BRAFi and MEKi, can result in complex interactions given the varied effects of these inhibitors on immune cells and tumor cells (Figure 7). In this study we show that treatment with MEK inhibitor consistently decreased PD-L1 mRNA and protein expression levels *in vitro.* Both thyroid cancer cell lines and tumor samples from patients with BRAF^*V600E*^-mutated tumors had higher levels of PD-L1 compared with either BRAF^*WT*^ tumors or matched normal tissue.

Accumulating evidence points to a relationship between MAP kinase signaling activation and the induction of PD-L1 expression [33, 38, 39]. Our study exploits the opposing effect of BRAFi on cells to prove this relationship. It has been reported that BRAF inhibitors block the MAP kinase signaling pathway in BRAF^*V600E*^ cells, whereas in BRAF^*WT*^ tumors, BRAFi activates the RAF-MEK-ERK pathway and could enhance tumor growth in xenograft models [40, 41]. Moreover, our lab previously showed persistently higher levels of phospho-ERK-1/ERK-2 protein when TPC-1 (BRAF^*WT*^) cells were treated with PLX4720 [28]. We blocked the hyperactive pathway in BRAF^*V600E*^ cells with BRAFi and MEKi and we activated and blocked the same pathway in BRAF^*WT*^ cells with BRAFi and MEKi, respectively. We demonstrated tight correlation between MAP kinase activity (as measured by ERK phosphorylation) and the expression of PD-L1 both in the mRNA and protein levels. IFNγ, a known and potent upregulator of PD-L1, abrogated the suppression that BRAFi imposed on PD-L1, but it was MAP kinase activity that consistently correlated with fine differences of expression as exhibited in our Western blot results.

The combination of BRAFi and immunotherapy is gaining acceptance as a rational approach to cancer treatment, and is undergoing clinical investigation in melanoma [42], where BRAFi has been shown to evoke tumor CD8^+^ T cell infiltration in melanoma patient samples [33, 43] and in thyroid mouse model [35]. Atefi et al. did point to the effect that BRAFi has on T cells when exposed simultaneously to PD-L1 [44]. These investigators showed that exposure of lymphocytes (which do not carry the mutant BRAF) to PD-L1 decreased activity of the MAP kinase pathway and lowered the production of cytokines. With the addition of vemurafenib (BRAFi), T cell-MAP kinase activity and cytokine production were restored. This surge of cytokine release, including IFNγ, has a potent effect on PD-L1 expression which in turn dampens the full potential of BRAFi on anti-tumor immunity.

Our previously established mouse model served as an ideal platform to explore the effect of PD-L1 blockade in the presence of BRAFi. On the basis of the above rationale, we hypothesized that a synergistic clinical and immunologic effect would be observed by combining both approaches. Indeed, our results showed dramatic tumor shrinkage when anti-PD-L1 Ab and BRAFi were combined compared to BRAFi alone. IHC analyses demonstrated an intense and dense infiltration of CD8^+^ T cell staining for cytotoxic marker. Moreover, the blockade of PD-L1 mitigated the effect of CD8^+^ T cells on the number of FoxP3 T regulatory cells in the tumor microenvironment. Multiple previous studies have shown that tumor-specific CD4+FoxP3 Tregs express multiple inhibitory receptors, including PD-1 and TIM3, which likely reduce the capacity of T cells in the tumor microenvironment for proliferation and cytokine production [45].

It is noteworthy that PD-L1 blockade and not BRAFi resulted in higher expression of the exhaustion marker, TIM3. This observation was not consistent with Fredrick et al., who reported increased expression of TIM3 with BRAFi treatment [33]. Ascribing the intensity of TIM3 solely to the intensity of the immune infiltrate would be far too simplistic. The intracellular signal that drives T helpers or CD8^+^ cells to over express TIM3 is not clear, but its co-expression with PD-1 on tumor specific CD8^+^ T cells makes it an important future candidate for combined immunotherapy [46–48]. Nevertheless, in the environment of PD-L1 blockade, increased TIM-3 and PD-1 expression may reflect the higher level of T cell activation rather than be an exhaustion marker. Given that the immune system, itself, does not appear capable of actively engaging and destroying metastatic deposits in lymph nodes in patients, testing combinations of therapeutics that inhibit the suppressive activity of Tregs may also be a fruitful line of investigation.

Our study does have a number of important limitations. Although the toxicity and tolerability profile of anti-PD-1/PD-L1 in clinical trials is promising [17, 49], our study was limited by the short duration of treatments as enforced by the rapid tumor growth in our model making it difficult to determine possible toxicity.

Adverse events (AE) related to anti PD-1/PD-L1 antibody as a single therapy and to a greater extent when combined with anti CTLA-4 or BRAFi are well documented in patients with melanoma and lung cancer [50, 51]. Most of the documented AE are low grade and include elevated liver function tests, pneumonitis and other immune-related adverse events that mainly involve the gut, skin, endocrine glands, liver, and lung. This combination of BRAFi and anti PD-1/PD-L1 was investigated in melanoma murine model with no documented treatment-related toxicity [30].

Since previous studies have not established whether tumors that have been growing slowly over time versus those that grow more aggressively, such as in our model, alter the native immune response to the tumor, it is not entirely clear where our model fits in the spectrum. In addition, genetically engineered tumor cell lines (such as ours) may have a smaller mutation burden compared to the equivalent native tumor. It is not entirely clear whether the immune response in the genetically engineered models is a true representation of the immune response in human tumors. Since thyroid cancers generally lie on the low end of the mutation burden, this might be less of an issue in thyroid cancer than other genetically engineered models used for preclinical studies [52]. Future murine tumor models with a longer pre-established therapeutic window for anti-PD-L1 will give better insight into survival patterns and perhaps a more accurate picture of its effect on tumor volume. In addition, failure of native T cells to clear tumors as they slowly grow is likely not entirely due to the PD-1/PD-L1 interaction. Here, we did not investigate the role of PD-L2, though in general it appears to have more restricted expression, is less prevalent across tumor types and thus does not appear as important in tumor's immune evasion [13]. However, exceptions do exist and in esophageal cancer, it appears that changes in PD-L2 levels driven by Interleukin-4 feedback loops, is a major part of the suppression of the immune response [53]. In our study, we also did not address possible significant impact of macrophages. Macrophage function is known to be a significant component of human ATC [54], and yet our previous analysis of our immunocompetent ATC model did not show a similarly large influx of macrophages; thus we did not further analyze the macrophage component in this study.

The fact that the study of tumor immunity has gone from a fringe effort to a viable and creative option in a wide range of tumors led us to take the first exploration in aggressive thyroid cancers. Our study is unique for its utilization of an immunocompetent *in vivo* model that permits exploration of the various players of antitumor immunity in the growing tumor. More than the exciting and robust immune infiltration that occurs when BRAFi and anti-PD-L1 work synergistically; it is obvious from the IHC results of our control group that aggressive thyroid tumors potentially grow without propagating anti tumor immune response. This observation, although hardly new, is striking given the robust immune infiltration when treatment with BRAFi and combinatorial therapies are implemented. Evidence from other malignancies, along with this preclinical study, suggests promise for strategies that combine immunotherapy with targeted therapy in thyroid cancer patients. The scarce number of aggressive thyroid cancer patients treated with BRAFi and the lack of *ex vivo* samples from these patients is a missing piece in the puzzle. We hope that in the near future, as clinical trials with targeted therapies and immune checkpoint regulators are developed for thyroid cancer, that pre and on treatment biopsy samples will be obtained from these patients to allow detailed analysis of the immune environment inside the tumors. This will allow a better understanding of the immune landscape of those who respond versus those who do not.

## MATERIALS AND METHODS

### Cell culture, reagents and antibodiess

Six human thyroid cancer cell lines; 8505c (Deutsche Sammlung von Mikroorganismen und Zellkulturen), BCPAP (previously provided by Dr. G. Damante of the University of Udine, Italy), SW1736, TPC-1 (provided by Dr. F. Frasca, University of Catania, Catania, Italy), HTh-7 and HTORi; four murine thyroid cancer cell lines; 3601R, 3868, 3743, 3403 [55], and four human melanoma cell lines; A375, A2058, UACC903 and MelJuso, were used in the study. All cell lines were cultured in Dulbecco's Modified Eagle's Medium (DMEM) supplemented with 10% fetal bovine serum and penicillin/streptomycin and incubated at 37°C in a 5% CO_2_ incubator. Cells were plated at 70-80% confluence and treated for 24 hours with either 10μM PLX4720 (Plexxikon, Berkeley, CA), 10μM PD03250901 (LC Biolabs, Woburn, MA), or 500U/ml hIFNγ for human cells and 200ng/ml mIFNγ for murine cells (BD Biosciences, San Jose, CA) either as single agents or in combination as specified in the results section. At the end of the treatment, cells were collected for RNA or protein isolation. Cell characteristics and mutational status are presented in Table 2.

**Table 2:** Cell line characteristics and their mutational status

*Cell line*	*Type*	*Important mutations and deletions*
**8505c**	ATC	BRAF^V600E/−^, P53 mutation
**BCPAP**	PTC	BRAF^V600E/WT^, P53 mutation
**SW1736**	ATC	BRAF^V600E/WT^, P53 mutation
**TPC-1**	PTC	BRAF^WT^, RET/PTC, PIK3A
**HTh-7**	ATC	BRAF^WT^, PTEN^−/−^, NRAS
**HTORi**	NT	BRAF^WT^
**A375**	MEL	BRAF^V600E/WT^, P53 mutation
**A2058**	MEL	BRAF^V600E/WT^, PTEN^−/−^, P53 mutation
**MEL-JUSO**	MEL	BRAF^WT^, RET/PTC, NRAS
**UACC-903**	MEL	BRAF^V600E/WT^, PTEN mutation

NT - Normal thyroid, MEL - Melanoma, ATC – Anaplastic Thyroid Cancer, PTC - Papillary Thyroid Cancer.

In preparation for these studies, a dose-response curve for hIFN-γ treatment was generated and a working concentration of 500U/ml, the minimum dose required to reach a saturated response for the 24-hour treatment, consistent with previously validated concentrations (data not shown), was selected [56].

phospho-p44/42 MAPK (ERK1/2) (Thr202/Tyr204) (phospho-ERK), p44/42 MAPK (ERK1/2) (Total ERK) and β-actin were purchased from Cell Signaling Technology.

The anti-PD-L1 antibody (10F.9G2) has been described previously [57], had less than 2EU Endotoxin per mg protein and was injected intraperitonealy (I.P) as described later.

### RNA isolation and real-time polymerase chain reaction

RNA isolation was performed using Trizol (Invitrogen, Carlsbad, CA), and the cDNA was synthesized using the Superscript VILO cDNA synthesis kit (Invitrogen) according to the manufacturer's instructions. PD-L1 gene expression was measured using TaqMan Gene Expression Assays (Invitogen, Primer i.d Hs-01125301_m1, Mm00452054_m1) by real-time reverse transcriptase polymerase chain reaction (RT-PCR) with technical triplicates and repeated at least twice. Gene expression levels were normalized to GAPDH expression.

### Protein extraction and western blot

At the end of treatment, cells were washed twice with PBS and lysed rapidly in RIPA lysis buffer containing protease and phosphatase inhibitor cocktails (Thermo Scientific, Rockford, IL) by incubating on ice for 10 minutes. Total lysates were centrifuged at 15,000 G for 15 minutes at 4°C, and supernatants collected and quantified using Bradford protein assay kit (Pierce, Rockford, IL). Western blot analysis was performed using standard procedure, and the proteins were identified using specific antibodies; total ERK (tERK), phosphorylated p42/44 ERK (pERK), β-actin and PD-L1.

### Orthotropic thyroid cancer mouse models of human ATC and PTC cell lines

All animal experiments were conducted at the Massachusetts General Hospital in accordance with federal, local, and institutional guidelines. To test PD-L1 expression and response to BRAFi therapy using human thyroid cancer cells: 24 ten-week-old female SCID mutant mice were injected with human thyroid cancer cells (12 with BCPAP and 12 with 8505c) as previously described [58]. Briefly, mice were anesthetized, the thyroid gland exposed, and 10^6^ cells injected into the left thyroid gland using a 27-gauge needle. The right side of the thyroid gland was not manipulated and was used as an internal control. Mice were randomized and started treatment at two weeks for the 8505 and 4 weeks for the BCPAP-implanted groups (see schema in Figure 4A). Six mice in each group received PLX4720-impregnated chow (Research Diets, Inc.) *ad libitum* while the other six were fed control diet (Research Diets, Inc.). After 14 days of treatment, mice were euthanized and both fresh and formalin-fixed tumors were collected for RNA isolation and IHC analysis, respectively.

### Immunocompetent mouse model of murine ATC using Anti-PD-L1 Ab

To test tumor growth and anti-tumoral immune indices in response to PD-L1 – PD1 blockade, with or without BRAFi therapy, we used the genetically engineered murine ATC cell line in an immunocompetent setting. First we implanted 10^5^ of the TBP (***T**POCreER; **B**raf ^tm1Mmcm/WT^; Tr**p**53^tm1Brn/tm1Brn^*) 3747 BRAF^*V600E/WT*^ P53^*−/−*^ murine tumor cell line in 24 immunocompetent syngeneic B6129SF1/J mice as previously described [29, 35]. In this immunocompetent model, the tumor progresses quite rapidly, and by 2 weeks the tumors have reached lethal size. Thus, one week after tumor implantation, the mice were randomized into 4 treatment groups. The control group received control chow (n=6), the PLX-treated group received PLX4720-impregnated chow (n=6), the anti-PD-L1 antibody group (n=6) received control diet and was injected with anti-PD-L1 antibody (10F.9G2) at 200ug/mouse/I.P dose every other day (4 total doses), and the combined treatment group received both PLX4720 chow and anti-PD-L1 antibody (4 total doses) (n=6). On day 14, all groups were euthanized and tumors collected. Tumor volume was calculated using the formula: (π/6) × length × width × height. tissue samples were collected and processed for IHC analysis [59].

### Immunohistochemistry

4-μm sections of the formalin-fixed orthotropic thyroid tumors were stained with Hematoxylin and Eosin stain and underwent IHC analysis for CD8 (Leica Microsystems), FoxP3 (eBioscience), TIM3 (Millipore), Granzyme B (abcam), mouse PD-L1 (clone 10F.9G2) (Provided by GF), and human PD-L1 (ProSci-Iinc Poway CA) primary antibodies. The primary antibody was detected using the respective biotin-free secondary antibody.

### Patient samples

Using the IRB-approved MGH Endocrine Surgery Tissue Bank and the Endocrine Surgery Data Registry, we obtained clinical data, fresh frozen tumors, and matched normal tissues from randomly selected patients with PTCs >1 cm. A total of 16 BRAF^*V600E*^ and 12 BRAF^*WT*^ tumors with their matched normal thyroid tissue samples were analyzed for PD-L1 mRNA expression.

### Statistical analysis

Statistical analysis was carried out using Microsoft Excel (Microsoft, Redmond, VA, USA) and IBM SPSS Statistics (version 23.0, IBM, Armonk, NY). BRAF mutant and wild type groups were compared using the Student t-test for age, Fisher's exact test for sex, tumor size (T1-2 vs. T3-4) and lymph node status, and Pearson chi-square for TNM stage. All experiments were performed in technical triplicate and repeated twice unless specified otherwise. A fold change in mRNA expression of 2 or more was considered significant. A log power of 2 for fold changes in expression was used to generate box-and-whisker plots based on the median and inter quartile range, and the nonparametric Mann-Whitney U- test was used to compare independent groups. Two-way factorial analysis of variance (ANOVA) was applied to test the effects of treatments on reduction in tumor volume with the Wald chi-square test to assess the additive vs. supra-additive (synergistic) effect of two-drug combinations. Two-tailed values of P < 0.05 were considered significant for all analysis.
